# Scotland and the Nurses' Registration Bill

**Published:** 1908-12-19

**Authors:** 


### Nursing Administration.

### SCOTLAND AND THE NURSES' REGISTRATION BILL.

MEETING IN EDINBURGH.

On December 10 a meeting to consider the application of this Bill to Scotland was held in the hall of the Royal College of Physicians, Edinburgh. It was attended by Dr. W. Allan Jamieson, President of the Royal College of Physicians, Edinburgh; Dr. Affleck, Dr. Playfair, Colonel Warburton, Superintendent of the Royal Infirmary, Edinburgh; Professor Glaister, President of the Faculty of Physicians and Surgeons, Glasgow; Dr. Mackintosh, Superintendent of the Western Infirmary, Glasgow; Dr. Maxtone Thom, Superintendent of the Royal Infirmary, Glasgow; Dr. Ker, Superintendent of the City Hospital, Edinburgh; Dr. Core, Superintendent of Stobhill Hospital, Glasgow; Dr. Richard, Medical Officer Govan Parochial Hospital; Dr. Newman, Glasgow; Dr. Johnston, Medical Superintendent of the District Hospitals, Glasgow; Dr. Brownlee, Superintendent of the Belvidere Hospital, Glasgow; Dr. Neil Carmichael; Miss Gill, Matron Royal Infirmary, Edinburgh; Miss Melrose, Matron Royal Infirmary, Glasgow; Miss Gregory Smith, Matron Western Infirmary, Glasgow; Miss Cowper, Superintendent of Queen's Jubilee Nurses; Miss MacFarlane, Matron Victoria Infirmary, Glasgow; Miss Merchant, Matron Eastern District Hospital, Glasgow; Miss Shepherd, Matron Govan Parochial Hospital, Glasgow; Miss Moseley, Matron Western District Hospital, Glasgow; Miss Wright, Matron Stobhill Hospital, Glasgow. Miss King, Miss Graham, and Miss Renwick represented private nursing institutions.

Dr. W. Allan Jamieson, who presided, stated that this meeting had been called in the interest of Scottish nurses in order that the provisions of the Registration Bill, in so far as they affect Scotland, should be carefully examined. Unity of feeling and action on the part of Scotland was necessary.

Dr. Mackintosh explained the need for united action, and proposed the appointment of a small Executive Committee to consider the provisions of the Bill in detail, and, if necessary, to suggest a scheme suitable for Scotland. As the Bill stands, Scotland is very inadequately represented on the proposed Registration Council. Although the present Meeting is highly representative of Scottish nursing, no person present is a member of the Matrons' Council, which clearly indicates that the Matrons' Council does not really represent the nursing profession. He strongly objected to the inclusion of "past" matrons in the Registration Council. Persons not engaged in active work soon lose touch with the profession, and it would be a mistake to pass over the numerous excellent matrons in active service and appoint persons who had retired. He observed that neither the Scottish nor Irish Local Government Boards are to be represented on the Council.

Colonel Warburton agreed with Dr. Mackintosh, and pointed out the vagueness of the term for which the provisional Registration Council were to hold office. Professor Glaister considered that the Bill as it stood was wholly unsuitable for Scotland. The Scottish representation on the Registration Council is inadequate. Some of the bodies named in the Bill have no title to representation. He objected to the implied power of the Registration Council to prescribe the work of the probationer nurses. It would, in his opinion, be fatal to register a class of nurses of inferior training, as proposed in the Bill. For these reasons he thought that Scotland should unreservedly oppose the Bill in its present form.

Dr. Newman stated that the nursing profession in Glasgow is opposed to the Bill as at present framed. He believed, however, that registration is inevitable, and is prepared to assist in promoting a Bill suitable for Scotland. He agreed that a small Executive Committee should be appointed.

Dr. Brownlee pointed out the hardship that would be inflicted on nurses trained in fever hospitals if the present Bill becomes law. Drs. Affleck, Carmichael, and Thom supported the proposal to appoint an Executive Committee, which was unanimously carried. A committee consisting of nine members was then appointed, and held its first meeting on Tuesday, December 15.

